# The Hospitals Problem

**Published:** 1920-08-28

**Authors:** 


THE PUBLIC WELL-BEING—(*continued*)

### The Hospitals Problem.

### REFLECTIONS ON THE GOVERNMENT BILL.

It is too early yet to offer anything like a detailed or final criticism of the Ministry of Health (Miscellaneous Provisions) Bill, a draft of which was published a few days ago. The writer has already heard many critical comments on the measure, not only from people directly interested in the hospitals, but from social workers who have given time and money to the relief of the sick and to the advancement of institutional treatment. The most important feature of the Bill is that it appears to establish an important principle which, unless adequate safeguards are forthcoming, will cause voluntary hospitals to pass into the virtual control of a bureaucratic State Department. The significant provision is that which empowers county councils or county boroughs " to contribute on such terms and conditions as may be approved by the Minister to any voluntary hospitals or similar institutions within their area." What does this mean? It seems to us to mean that the Ministry of Health is inserting the thin end of the wedge of State control. If that is the object of the Government it is the worst method of approach imaginable. It leaves nominal control with the hospital authorities, but vests the real underlying control in the Ministry of Health, acting through the public bodies. It may also have the serious effect of drying up the main sources of hospital revenue by alienating the sympathies of voluntary subscribers, who will not be disposed to advance their money in relief of the rates, and many of whom regard with dismay the prospect of a State-run hospital system.

It is quite true that owing to the increased cost of living and the greatly enhanced cost of the administrative services in our charitable institutions, both the London and provincial hospitals are

**THE PUBLIC WELL-BEING—(*continued*).**

in urgent need of assistance—not merely of a special public effort, but of a general and continuous raising of the basis of contributions. They have become so vital a part of the well-being of the nation, and have developed into such great and complicated concerns, that it is impossible to suppose they will be able to survive on their present lines if they have to depend on a spasmodic, haphazard, and hand-to-mouth existence. Indeed, writing a few weeks ago, Lord Knutsford frankly admitted that State assistance is absolutely necessary if the London Hospital and all the other great hospitals are to remain open. But what kind of State assistance? And will State assistance mean the break-up of the voluntary system, which stands for so much that is generous and humane and unselfish in the life of the nation? *The Times* has suggested that the situation might be met by means of an emergency grant, but the crux of the matter is that it is not only the immediate situation that has to be considered, but the whole problem of the financial future. If the hospitals could rely upon an adequate grant regularly forthcoming, or upon deficiencies not due to inefficient administration being made up from year to year, a great stimulus would be given to the cause of the sick and suffering. On the other hand, this financial benefit would have to be answered by an effective co-ordination of effort among the voluntary hospitals, and a still closer supervision of their manifold activities.

One provision in the Bill is excellent. It allows the county authorities to establish, or maintain, or to contribute to the cost of, the ambulance services. These are essentially public services, and the public ought to pay for them. The proposal, which might be carried beyond an optional arrangement, in itself suggests how relief by the State can be extended through assistance to such important, though subsidiary, hospital functions as medical education and medical research. We shall no doubt hear a great deal more about these various possibilities when the Bill is debated in Parliament.

